# End-to-End Deep Learning by MCU Implementation: An Intelligent Gripper for Shape Identification

**DOI:** 10.3390/s21030891

**Published:** 2021-01-28

**Authors:** Chung-Wen Hung, Shi-Xuan Zeng, Ching-Hung Lee, Wei-Ting Li

**Affiliations:** 1 Department of Electrical Engineering, National Yunlin University of Science and Technology Yunlin, 123 University Road, Section 3, Douliou, Yunlin 64002, Taiwan; wenhung@yuntech.edu.tw (C.-W.H.); m10912045@yuntech.edu.tw (S.-X.Z.); etaowtli@auo.com (W.-T.L.); 2 Department of Electrical and Computer Engineering, National Chiao Tung University, 1001 University Road, Hsinchu 300, Taiwan

**Keywords:** convolutional neural network, vibration signal, short time Fourier transform, shape identification, MCU

## Abstract

This paper introduces a real-time processing and classification of raw sensor data using a convolutional neural network (CNN). The established system is a microcontroller-unit (MCU) implementation of an intelligent gripper for shape identification of grasped objects. The pneumatic gripper has two embedded accelerometers to sense acceleration (in the form of vibration signals) on the jaws for identification. The raw data is firstly transferred into images by short-time Fourier transform (STFT), and then the CNN algorithm is adopted to extract features for classifying objects. In addition, the hyperparameters of the CNN are optimized to ensure hardware implementation. Finally, the proposed artificial intelligent model is implemented on a MCU (Renesas RX65N) from raw data to classification. Experimental results and discussions are introduced to show the performance and effectiveness of our proposed approach.

## 1. Introduction

Shape identification of workpiece objects is an important issue in industrial applications due to high rate of production and automatic system efficiency. There are many references that discuss workpiece sorting [1,2,3,4,5,6,7,8]. For tube material applications, the geometric features are measured by automated optical inspection technology then machine learning models, e.g., neural network (NN), support vector machine, and random forest, were used for sorting or identification [3,4]. Reference [5] introduced a flexible machine vision system and a hybrid classifier for small part classification. However, these studies are all machine vision approaches and the system costs are high due to the use of high resolution cameras and lenses. Recently, other articles have adopted clamped force for object classifiers [6,7,8]. Reference [6] considers the natural frequencies of materials and uses a NN to perform non-destructive testing. In [7], the signal data from force and position sensors are used in the proposed K-means clustering algorithm to identify grasped objects. Reference [8] presents a classification method by sliding the robotic fingers along the surface and the K mean-neural network classification was used. In several applications, the classification of grasped objects was accomplished by using mechanical properties of objects, e.g., damping coefficient, mass, spring; however, these parameters need force and position sensors [7,9,10]. To address these issues this paper introduces a low cost intelligent gripper for sharp identification by vibration signals. Two MEMS accelerometers are utilized to collect vibration raw data and an on-line deep learning algorithm is implemented by a MCU.

There are many references that address data preprocessing methods, such as discrete wavelet transform and crest factor [11,12,13,14,15]. In general, a fast Fourier transform (FFT) is selected to preprocess and the frequency features are used for machine learning [12,13,14,15]. Different from standard Fourier transform to obtain a spectrum of fully time domain samples, while short-time Fourier transform (STFT) is a sequence of Fourier transforms for short intervals [16]. Then, the spectrum variation of samples over time is kept in STFT. The STFT is used to transfer vibration signals into images for classification here. Feature extraction and selection are other issues in machine learning classification. Many feature extraction methods have been proposed using principal components analysis (PCA) [13,17,18], autoencoders (AEs) [18,19,20,21,22], and convolutional neural networks (CNNs) [23]. PCA requires less calculation and has faster responses and AE is suitable for situations lacking negative sampling. Recently, CNN has been indicated to be better for feature extraction of images. STFT and CNN are used in fault diagnosis of bearings and also implemented for gearbox faults diagnosis [24,25]. Here are some methods based on STFT and CNN for classification, such as the flutter in the aircraft [26], and ECG arrhythmia classification [27]. As mentioned previously, STFT preprocessing provides frequency information of signals but also tracks their variation over time. The STFT creates time-frequency domain information and the CNN is suitable for feature extraction of input signals.

**Figure 1:**
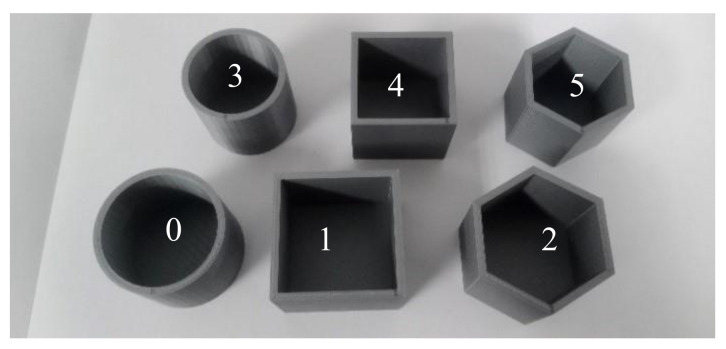
The candidate objects.

This paper proposes a real-time approach for shape identification by deep learning. A pneumatic gripper is embedded with two accelerometers to sense acceleration (vibration signals) on the jaws for identification. The raw data is firstly transferred into images by STFT and then the CNN is adopted to extract the features for classifying objects. The whole model is implemented in a micro-computer unit (MCU), and all calculations are also accomplished in this MCU. The candidate objects identified by the proposed system are shown in Figure 1. There are six sorts with 2.3 mm of thickness, which are square column, cylinder and hexagonal columns with two radii (40 and 35 mm). These six sorts are large-cylinder, large-square column, large-hexagonal column, small-cylinder, small-square column, and small-hexagonal column, and are indexed with the numbers 0 to 5. In addition, the AE approach with FFT inputs is also introduced to establish an identification system for other objects. Several experimental results and a discussion are introduced to demonstrate the performance and effectiveness of our approach.

As mentioned above, the STFT image and CNN are utilized for development of our model which is implemented on a MCU. However, the limitation of MCU memory will affect the accuracy. Therefore, the hyperparameters of the model are important for practical implementation. Some procedures are discussed to decide the best hyperparameter, such as random search (RS) [28] and grid search (GS) [29]. The former may generate good hyperparameters faster, but optimization is not guaranteed. GS only focuses on a limited searching range and does not require more calculation time, however, it can achieve the optimization and provide better reproducibility. Some bias may be induced in the model when feeding any one particular division into test and train its components. The training or test data will affect the training result. To avoid this situation, cross validation is used [22,29,30]. Moreover, an early stopping scheme is also adopted when searching for the best hyperparameters, which prevent overfitting [31,32]. Herein, GS and cross validation are also used for the hyperparameter optimization to guarantee efficiency and performance.

This paper is organized as follows: Section 2 introduces the problem definition and the proposed system description. The proposed intelligent gripper system is introduced in Section 3. The experimental results are introduced in Section 4. Finally, the conclusions is given.

## 2. Identification Approach by Deep Learning

### 2.1. Signal Prepocessiong

**Figure 2:**
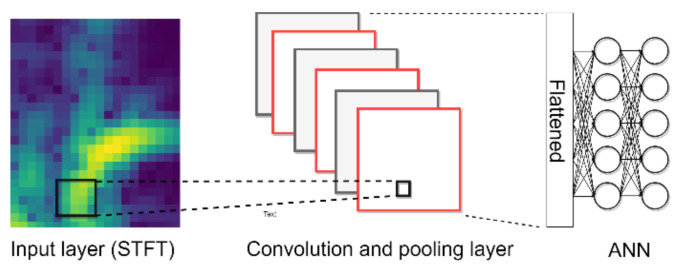
STFT-CNN for shape identification.

In this paper, on-line vibration signals are adopted to establish an intelligent gripper using a deep learning approach. Figure 2 shows the proposed intelligent approach. The vibration signals are first transferred into image by STFT and the CNN is adopted for accurate identification. STFT is a method to not only to present frequency information, but also to provide the frequency variation in time-localization. The time domain data will be converted into a two-dimensional image of the time and frequency domain. The STFT time-frequency spectra of signals are generated using the following expression:

(1) $$s_{k,m,l}=\sum_{n=0}^{N-1}x_{n,l}\times \omega_{m-n}\times e^{-j\frac{2\pi k}{N}n}$$

where *s* indicates the values in frequency domain, *k*, *m,* and *l* are the indexes of the frequency, time window, and input channel; *x* presents the sampling data in the time domain and *n* indicates the total number of sampling points; ω indicates the window function and *n* indicates the offset in the window function. The number of input channel depends on the number of channels used in system. As mentioned above, two *x*-axis-accelerometers are used to develop the proposed system, whereby two acceleration channels are sampled. Next, the sampled acceleration is transformed into a time-dependent frequency type, function then these data are feed to the CNN for feature extraction.

### 2.2. Convolution Neural Network

**Figure 3:**
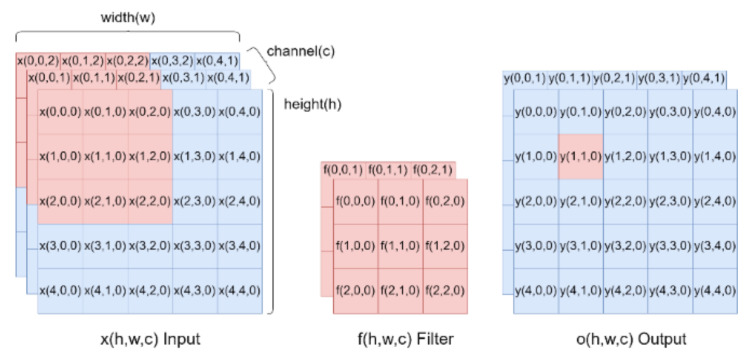
Illustration of convolutional operation.

The CNN has recently become a popular neural network for image processing and it is used in this proposed system for the feature extraction and identification task. Convolutional and pooling operations in a CNN can help to extract the features of continuous arrays. The feature maps after extraction are flattened and applied as inputs of fully-connected layers for further applications. Figure 3 shows the convolution operation, where the left part is input data (*h* × *w* × *c*), *h* indicates points in the height-axis, w is the width-axis, and c is the input channel number; the middle part is the feature detector filter (*h_f_* × *w_f_* × *c_f_*); and the right part is the output (*h_o_* × *w_o_* × *c_o_*). The feature detector filter is taken to step through the input and map this filter one by one to extract a feature map. The calculation expressed in Equation (2) is called convolution, where *y* is the feature map and *h* and *w* indicate the *x*- and *y*- coordinate of the pixels, c is the output channel number; *h_f_* and *w_f_* are the filter size. The training process is used to search for the optimized parameters:

(2) $$y_{h,w,c}=\sum_{m=0}^{h_{f}}\sum_{n=0}^{w_{f}}x_{h-1+m,w-1+n,c}\times f_{m,n,c}$$

where *f* = 0, 1, 2, …, *h_f_* − 1 and *m* = 0, 1, 2, …, *w_f_* − 1.

**Figure 4:**
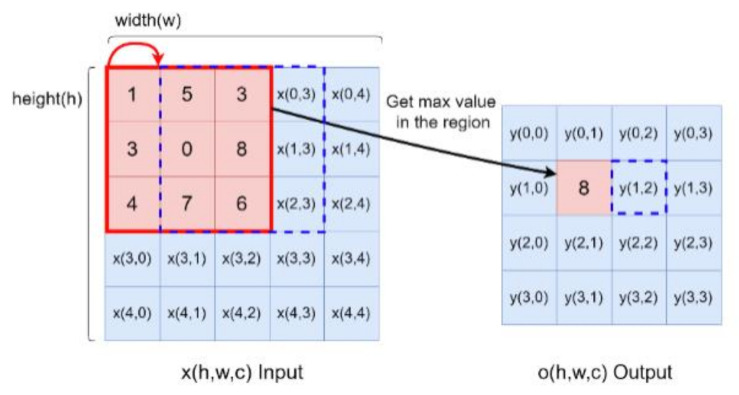
Max pooling operation illustration.

Max pooling is used for down-sampling after the convolution operation. The input size is (*h* × *w*), where *h* indicates points in the height-axis, *w* is the width-axis and the output data is (*h_o_* × *w_o_*). The max pooling kernel is 3 × 3 as shown by the red block in Figure 4, and the step number of the pooling is 1. At first, the maximum of a region is filtered as output data in pooling processing. Next, the mask will move from the red block to the blue block. After feature extraction, the two-dimensional features are transferred into a one-vector by flattening, then the fully connected layer is an artificial neural network (ANN), that plays the role of classifier. The operation is:

(3) $$y_{n}=\sigma (\sum_{m=1}^{M}(x_{m}\times w_{m,n})+bias_{n})$$

where *y* and *x* indicate the node values of the current and previous layers, respectively; *σ* is the activation function, *w* is the weighting between the layers and bias shows the bias. *M* is the node amount of the previous layer and *n* is the index of the current layer. The training process will find the most suitable values for the weighting *w* to perform the similar neural network function.

### 2.3. Autoencoder

**Figure 5:**
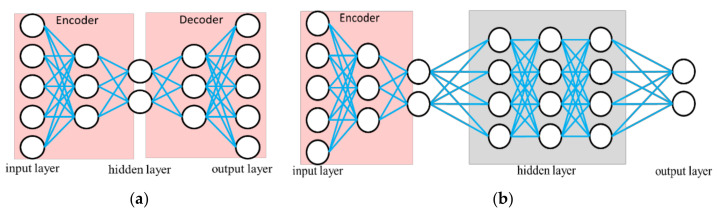
Illustration of autoencoder, (**a**) autoencoder for feature extraction; (**b**) autoencoder-NN for classification.

The AE is a type of NN used to learning efficient data coding in an unsupervised manner. The purpose of AE is to learn a representation for a set of data; it contains an encoder and a decoder, as shown in Figure 5. AE can be viewed as a neural network architecture with a bottleneck which forces a compressed information representation of the original input. That is, the encoder tries to learn a function that compresses the input into low dimension space (feature extraction). The decoder aims to reconstruct the input from this latent space represenation. The operations of a hidden layer are the same as in Equation (3). In addition, the corresponding loss function is the mean squared error (MSE) of the output. After training, the bottleneck layer provides the extracted features. Subsequently, the antoencoder-NN shown in Figure 5b is established for classification by supervised learning. This AE has the ability to reduce the complexity of the classification. The reconstruction error is also calculated from the difference between the original pattern and the decompressed output pattern. This mean that the reconstruction error can be used to detect defects. This model is used in many applications requiring yield detection and device detection [12,13]. Thus, we combine the proposed STFT-CNN and AE to develop a modified model for identifying other objects. Details will be introduced in Section 3. 

### 2.4. Hyperparameter Optimization

As indicated above, the STFT image and CNN are utilized for development of our model which is implemented on a MCU. However, the limitation of MCU memory will affect the accuracy. Therefore, the hyperparameter selection for the model is important for implementation. For realization of the system, the MCU is considered as the system platform, which means we have limited memory and calculation resources. The objective of hyperparameter optimization is not only accuracy but also the model memory size. In fact, in the proposed method, the latter may be higher priority.

Herein, the Tensorflow method is utilized to construct the machine learning model [33], and grid research and cross validation are used to evaluate the model accuracy for hyperparameter optimization. These better models obtained after a grid search and cross validation will be stored temporarily. The e-AI Translator supported by the Renesas software [34,35] are utilized to translate these models (.pb or checkpoint) into the C-language code which is used in the MCU, and the memory size and amount of calculation required by the machine learning model are also estimated. This estimation includes the read-only memory, ROM; random access memory, RAM; and the multiply and accumulation number (MAC) operations for calculation. For implementation, these three parameters are considered for model selection.

**Figure 6:**
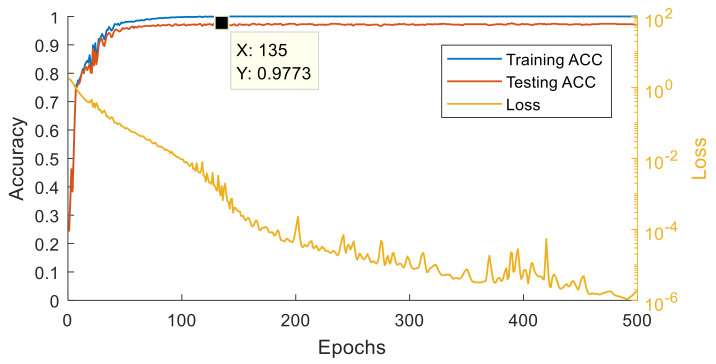
Training and testing results of AE.

The optimized parameters in the grid search are the filter dimension and step; channel number in CNN; the max pooling using; the node and layer number in the fully connected layer, ANN, as listed in Table 1, and the model size. Epoch time is set to 500 and the learning rate is 0.0002. To avoid any bias caused by random initial weights when training, every parameter combination will be trained three times and the results averaged. For each object, the gripping acceleration of 660 records is sampled. Thus there are in total 3960 records used for training, validation and testing. The data numbers of the training, validation and test dataset are 2400, 1200, and 360, respectively. The MCU used in this paper is a Renesas RX65N, it supports two million bytes of ROM and one million bytes of RAM. The model choice after GS and CV hyperparameter optimization has to consider the memory limitation. If the memory required by the best model is beyond the MCU specification, it will be skipped and a smaller model is considered. The optimization results are shown in Table 2. In addition, the structure of the AE algorithm, shown in Figure 5a, is also optimized, the corresponding training and optimized results is shown in Figure 6 and Table 3.

The memory requirements of all models are shown in Table 2 and Table 3, and indicate all could be supported in the chosen MCU. The MAC of the model ranked in the third place is smallest and its execution time is shortest. Compared with the fifth model, the first four models require similar and small calculation resources. The first model will be adopted to be implemented, since the RX65N MCU could support the memory requirements of the model. 

## 3. End-to-End Implementation by the MCU

### 3.1. System Description

**Figure 7:**
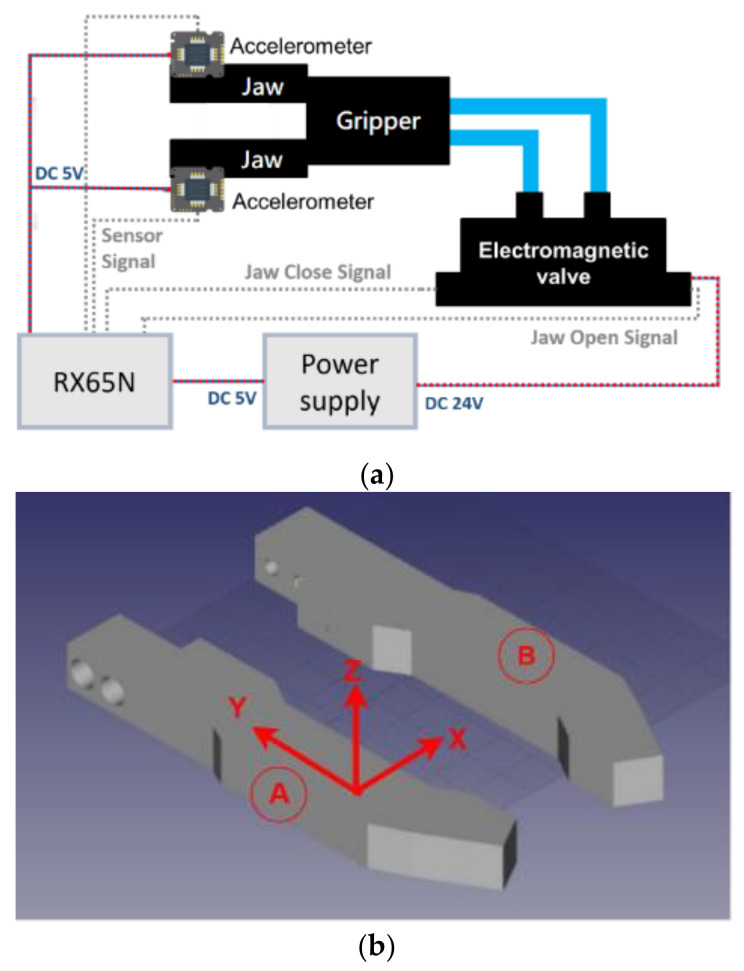
System description of the proposed system, (**a**) system diagram; (**b**)The detailed positions of accelerometer installation of jaws.

Figure 7 shows the proposed structure of intelligent gripper using deep learning, it includes a MCU (Renesas RX65N) [34], electromagnetic valve, gripper and power supply. The MCU is used to implement the proposed artificial intelligence identification approach. The electromagnetic valve is used to control the pressurized air for the action of the gripper jaws. The power supply is necessary for the 5 V and 24 V DC sources. Finally, the proposed object shape identification system is based on the acceleration information that is sampled by the accelerometers what are installed on the two jaws. The details of the positions of the accelerometer installation are shown in Figure 7b. Due to a lack of feedback information in the pneumatic gripper, an accelerometer is used to collect the dynamic variation for the object identification. The 3-axis accelerometer used in this paper is a MMA7361L [36], a low power, low profile capacitive micromachined accelerometer featuring signal conditioning, that could sample the acceleration of all three axes, so it could be installed on either the A or B side. However, only the *x*-axis acceleration is considered to perform the shape identification. If a one-axis accelerometer is selected, the installation position and direction need to be considered to capture the *x*-axis acceleration information.

**Figure 8:**
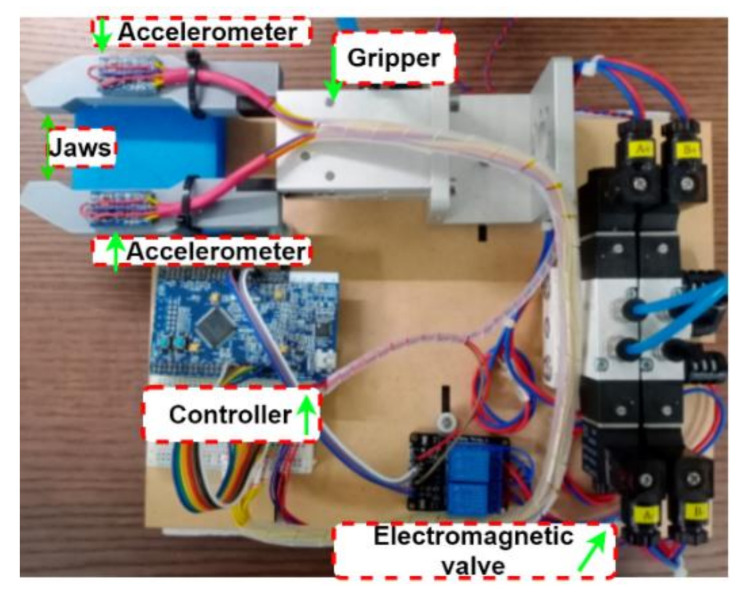
The realization of the proposed system.

The realization of the proposed system is shown as Figure 8. The Renesas RX65N MCU is used as the controller. It supports the peripheral function to generate the gripper control signal feeding to the electromagnetic valve and the analog to digital converter for acceleration sampling. All of AI identification algorithm is also implemented in the MCU based on hyperparameter optimization. The accelerometers are installed on the jaws for measuring the acceleration on the jaws when gripping. The MMA7361L accelerometer performs the conversion from acceleration of 0 to 6 G into an electric potential of 0 to 3.3 V proportionally.

**Figure 9:**
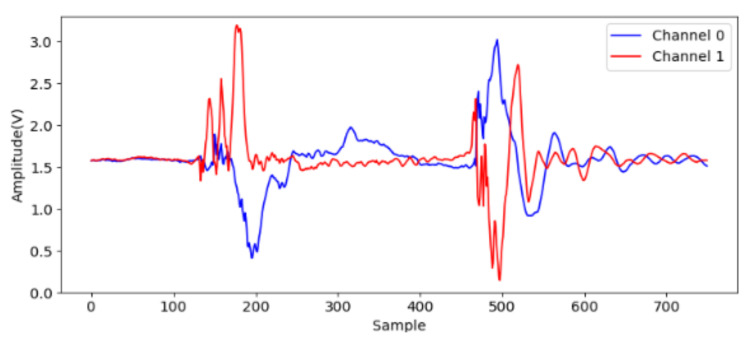
The acceleration voltage signals for no gripping.

**Figure 10:**
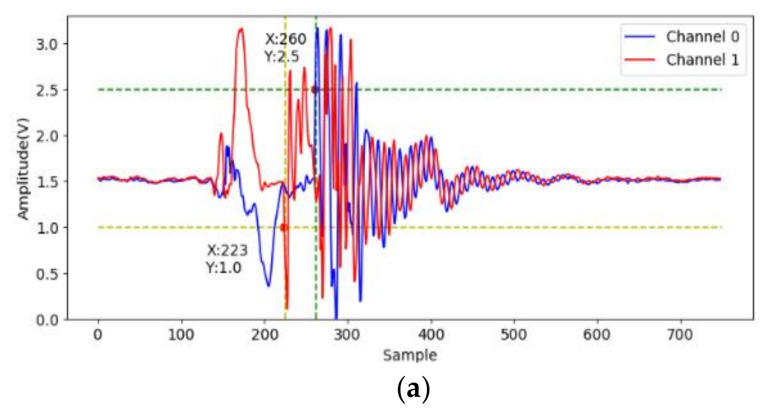

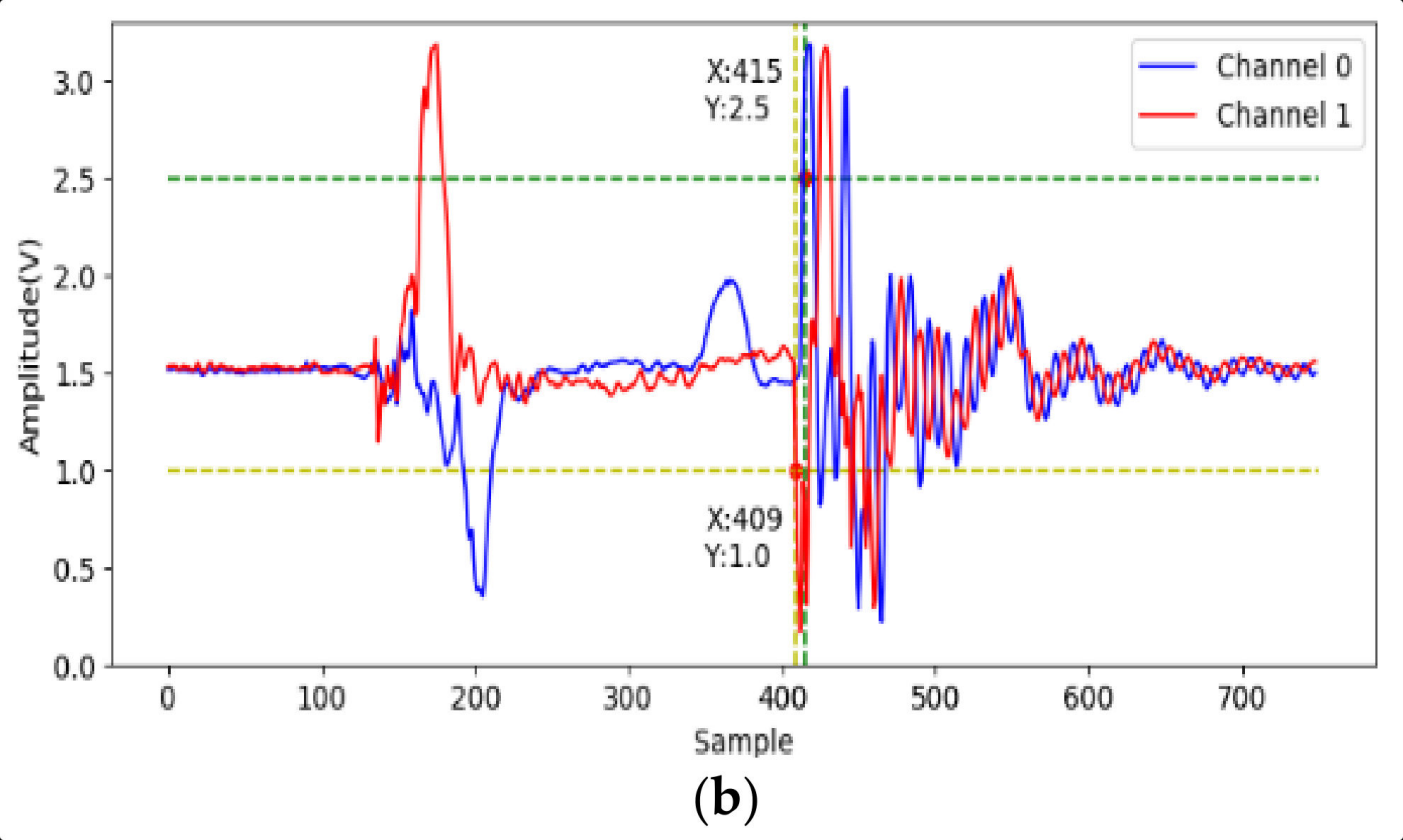
The acceleration voltage signals cylinder gripping, (**a**) large one; (**b**) small one.

At first, to identify effective signals from the acceleration sampling data, the acceleration when gripping six objects are sampled and analyzed. The two accelerometers measure the *x*-axis acceleration on the two jaws, and the channel 0 and 1 indicate lower and upper jaws, respectively. The data (750 points) are sampled on each channel at 7.5 kHz for 0.1 s after the valve is opened. We note that the gripping acceleration-voltage is sampled when no object is gripped and the corresponding data are shown in Figure 9. It can be observed that there is some variation from the 140-th to the 220-th points (18 ms to 29 ms). This noise may be caused by some mechanical error of the pneumatic gripper. The next variation around the 500-th point should be caused by the jaw contact. Obviously, the samples in this time range are useless and should be skipped. Figure 10 shows the acceleration-voltages when a large-cylinder and small-cylinder are gripped, and we can observe that there is some large variation when the jaws contact the objects. Two threshold values are set as triggers in the proposed identification system. When both threshold values are achieved, the trigger condition is established. It means both jaws contact the object, and the system captures the pre-trigger and post-trigger acceleration information from both two channels. From observation and our experiences, the threshold values are 2.5 Volts and 1 Volt for channel 0 and 1, respectively. Comparing Figure 10a,b, the trigger timing in the small-cylinder case is later than in the large-cylinder case, and apparently, the proposed trigger condition is useful to separate effective data from noise.

**Figure 11:**
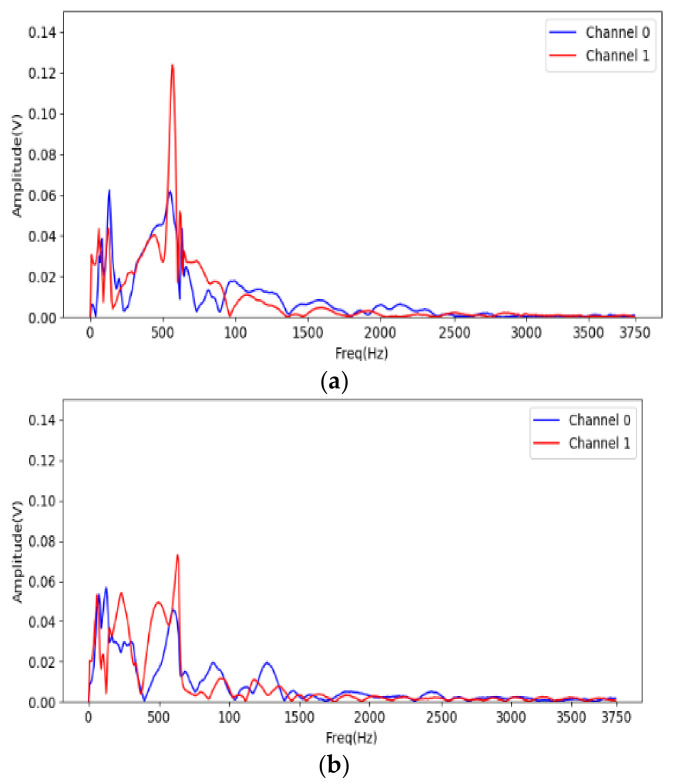
FFT analysis of cylinder gripping signal, (**a**) large one; (**b**) small one.

To recognize the useful frequency region for shape identification, FFT is used to analyze the acceleration on the jaws when gripping. The system starts to sample the acceleration for 1024 points at 7.5 kHz after triggering. The data are translated into 512 discrete frequency domain features. Figure 11 shows the spectrum of the acceleration on the two jaws for the large-cylinder gripping. Most of the acceleration frequency information is located under 1.5 kHz, therefore, only the STFT result from 0 to 1.5 kHz is fed into the CNN for identification.

**Figure 12:**
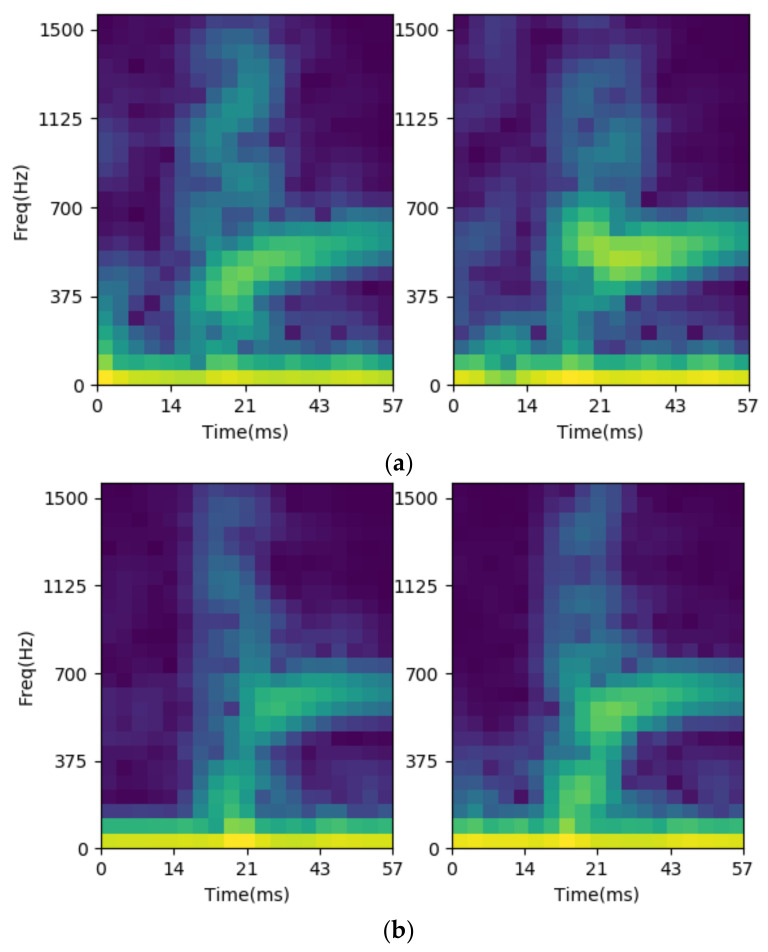
The STFT result for gripping small-cylinder, (**a**) large one; (**b**) small one.

As previously mentioned, STFT is utilized to preprocess the vibration signals into images in the proposed system. The FFT length is 128, the window function is Blackman-Harris. The frequency resolution is about 58.594 Hz, and only 25 points of spectrum representing less than 1.5 kHz is necessary. The number of overlapped samples is 112, which means the window function step is 16 points for each spectrum. For the sample completeness, the pre-trigger and post-rigger sample numbers are 176 and 256, respectively, and the total input of the STFT is 432 samples. There are 20 time-dependent-spectra in each channel and the data number is 25 points in each spectrum. Due to the use of dual channels, there are 1000 preprocessing data for feature extraction. The STFT results are shown as Figure 12 for cylinder cases, where the left/right figures are the STFT results of channels 0/1. We note that no matter the channel, 0 or 1, the spectrum at the beginning contains more rich information for the large-cylinder than the small-cylinder. This is the reason why the STFT results of the acceleration on the jaws could be used for identification.

### 3.2. Experimental Results and Discussion

#### 3.2.1. Experiment 1: Performance of STFT-CNN

**Figure 13:**
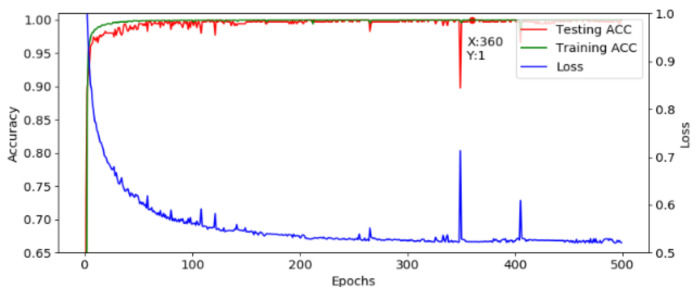
Training and testing results.

After optimization, a fixed structure is used to construct the developed AI model. The corresponding training result is shown in Figure 13, where the loss is decreasing and the accuracy is increasing and the model is converging normally. The highest accuracy is achieved at the 360th epoch, and the accuracy is 100%. Although the accuracy drops at the 349th epoch, it recovers fast. Moreover, the loss is flat between the 355th to 370th epoch, which shows that the model workable. This model is translated into the C language and implemented in the MCU.

**Figure 14:**
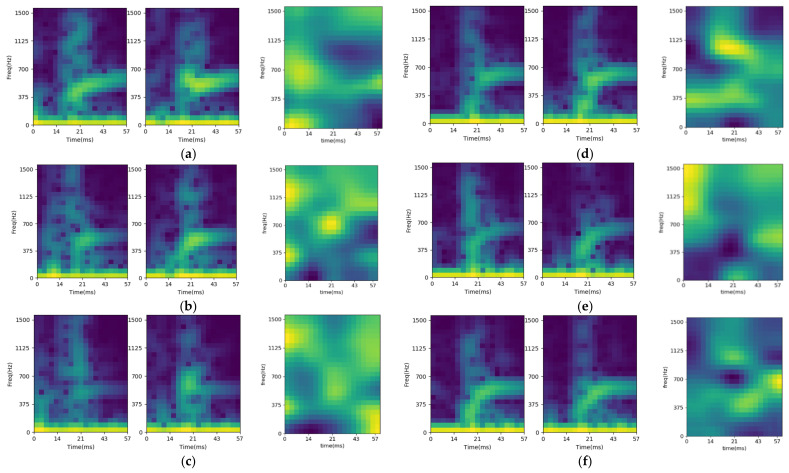
STFT input feature and activation map for (**a**) large-cylinder, (**b**) large-square column, (**c**) large-hexagonal column, (**d**) small-cylinder, (**e**) small-square column, and (**f**) small-hexagonal column.

To validate the feasibility, the training model is analyzed. For the CNN identification model, the Gradient-weighted Class Activation Mapping (Grad-CAM) method is used to provide visual explanations for the important regions of input [37,38]. The analysis results are shown as Figure 14, including the STFT input features and the activation maps of six sorted objects. The figures are the average results of 100 repetitions for each object. Comparing the activation maps of the large and small objects, the identification for small objects relies on the STFT features of the post-trigger spectrum regions (after 14 ms). On the contrary, there are some high activation regions located on the pre-trigger region (before 14 ms) for large objects. The feasibility of the model is verified by the existence of only a few high activation overlap regions.

In order to further evaluate the feasibility and reliability of the proposed system, an online test is necessary. Online tests of 100 times are made for each category and the accuracy of the results is 99.83%. The confusion matrix analysis is presented in Table 4. There are some misjudged cases between the large-cylinder and the small-cylinder, this error should be caused by the similar structures of the two objects. However, the true-positive rate (TPR), precision (PRE), and accuracy (ACC) achieve more than 99%. The proposed identification system works successfully. To illustrate the effectiveness of STFT-CNN, AE with FFT inputs are done for comparison. The corresponding FFT (512 datapoint) of vibration signals (1024 values) are obtained to be the inputs of AE. The optimized structure is introduced in Table 3. After feature extraction by AE, the NN model is established for classification by using the extracted features. We obtain an average ACC of 98.61% and an on-line test ACC of 95.6%.

#### 3.2.2. Experiment 2: Discussion on Location Variation of Objects

**Figure 15:**
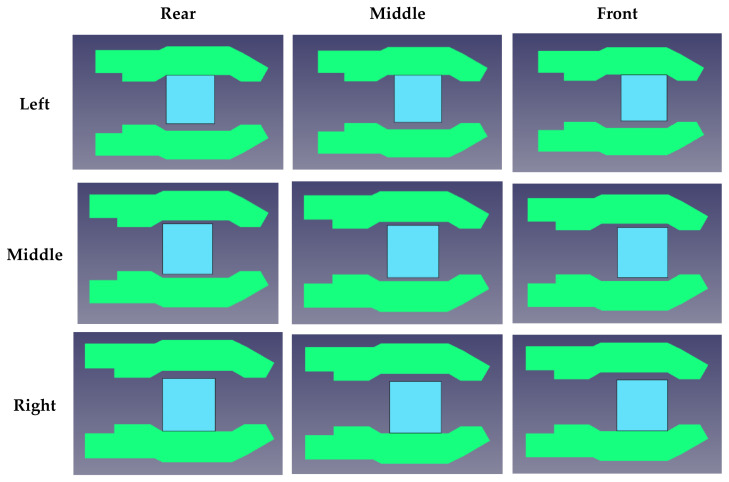
Illustration of the effect of object location.

**Figure 16:**
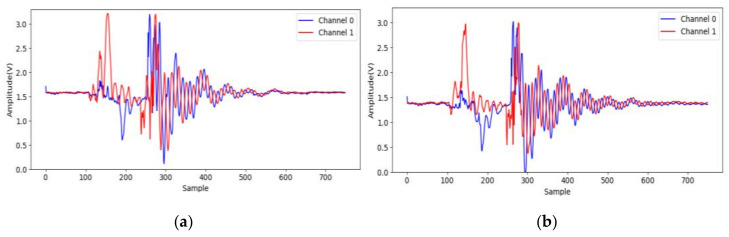
Acceleration signals of different locations for a large hexagon: (**a**) rear, middle; (**b**) middle, middle.

Herein, a demonstration experiment is introduced to observed the effect of the variation of object locations. The objects are located randomly in the region of the gripper (45 mm × 45 mm), shown in Figure 15. The corresponding acceleration signals of different locations for a large hexagon are shown in Figure 16, where we can observe the signal variation in both channels 0 and 1. The results of STFT-CNN trained by experiment 1 is 75% of ACC, so the location indeed influences the classification result.

**Figure 17:**
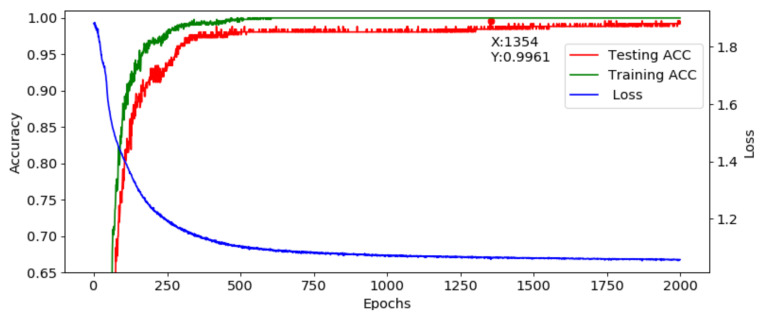
Training and testing results for location variation.

To ensure the robustness of the model, raw data of each locations shown in Figure 15 are collected for the training process (70 trials × 9 locations, total number of data 3780, 2700 data for training and 1080 for testing). The STFT-CNN is optimized and trained again. Figure 17 shows the training and testing results and the corresponding confusion matrix is introduced in Table 5. It can be found that the STFT-CNN performs well to obtain an ACC of 97.55%. This shows that the STFT-CNN in MCU is well designed if the collected data is enough.

#### 3.2.3. Experiment 3: Discussion for Classifying Other Objects

**Figure 18:**
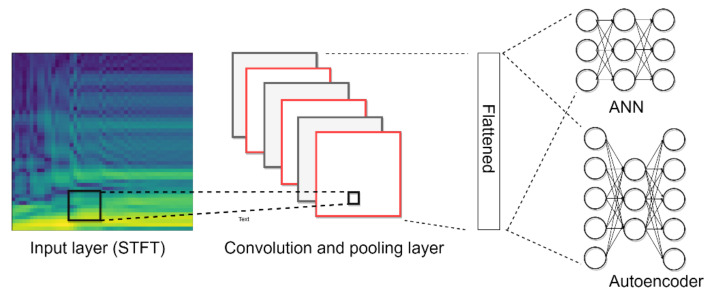
Combination of STFT-CNN and AE in parallel scheme.

We here to discuss the classification of pre-defined objects and other objects. Additional trapezoid and ellipse shapes are utilized for other shape labelling. For supervised learning model development (STFT-CNN), the objects should be labeled and pre-defined. This means that the model cannot identify other undefined objects. According to the description in Section 2.3, the AE has the ability for detect other undefined objects using the variation of reconstructed errors. Herein, we combine the STFT-CNN and AE in parallel scheme, shown in Figure 18, to treat the problem. An AE with structure (20-16-12-9-15-9-12-16-20) is connected after the flattened layer, and utilized for the estimation of other shapes. The reconstructed error is calculated by the mean square error (MSE) of the output and the value of 0.009 is adopted for the threshold. This means that the object is first judged by the reconstructed error and STFT_CNN. The corresponding rule can be introduced as follows: “If the reconstructed error is larger than the threshold, then the object belongs to another class; otherwise the object is one of the six predefined classes.”

The modified model is trained by data of six sorts and the corresponding confusion matrix is introduced in Table 6. We can observe the trapezoid and ellipse shapes can be estimated by the modified model and the total ACC is 98.5%.

#### 3.2.4. Experiment 4: Discussion for Thickness of Objects

Herein, the influence of object thickness is discussed. It is obvious that the thickness of objects results in different mechanical properties, therefore, the corresponding vibration signals are changed. Herein, six more sorts of samples with 4.6 mm of thickness are created. As in Experiment 2, we first adopt the model established by Experiment 1 for classification. We obtain results with TPR 31.5% and PRE 23.5% and most of them are classified to be square columns. By using the modified model shown in Figure 18, we obtain the classification results listed in Table 7. We can observe that thicker objects can be estimated by the modified model and the total ACC is 99.42%.

According to the previous experiments 1–4, we can conclude that the proposed approach has the ability for classification by using vibration signals if the objects are pre-defined. In addition, the proposed modified model by combination STFT-CNN and AE has the ability for classifying objects without being pre-defined.

#### 3.2.5. Experiment 5: Performance Discussion of Network Structure in MCU

Herein, the discussion and comparison of the network structure in the MCU is introduced. In Experiment 1, the total end-to-end time spent is 0.352 s with sampling time: 0.1 s; computation effort of STFT: 0.082 s, and AI model for classification: 0.16 s. Experiment 2 utilized a larger structure in which takes 1.23 s for classification, this results in 1.422 s for end-to-end classification. In addition, the experiments spend 0.818 s for one object classification due to the fact more features were selected (resulting in 0.590 s for STFT-CNN and 0.036 for AE). We summarize these results in Table 8.

## 4. Conclusions

This paper has introduced an end-to-end real-time classification approach from raw sensor data. The MCU-based implementation of an intelligent gripper is established for shape identification of grasped objects by CNN. To ensure the implementation, the CNN structure is optimized by grid search and cross validation. A pneumatic gripper equipped with two accelerometers is used for collecting vibration signals for identification. The raw data is firstly transferred into images by STFT and then a convolutional neural network is adopted to extract features and classify six sorts of objects. Objects of six different shapes and two sizes could be identified, and the accuracy was more than 99%. Only two accelerometers and a MCU are adopted, the AI model is implemented in the MCU for identification. The system is fully modularized and suitable for installation on automatic systems. Experimental results were presented to demonstrate the performance and effectiveness of our proposed approach. In addition, we can conclude that the proposed approach has the ability for classification by using vibration signals if the objects are pre-defined. In addition, the proposed modified model by combination STFT-CNN and AE has the ability for classifying objects without being pre-defined. In the future, the electric gripper with force estimation can be considered for classification by mechanical properties and vibration signals. The correlation between them can be discussed for simplifying judgement costs.

## Figures and Tables

**Table 1:** Grid Search Range Setting of Hyperparameters.

Hyperparameters	GS Range Setting
CNN channel	2, 4, 8
CNN kernel	3, 5
CNN filter step	1, 2
Max pool use	Use or not
ANN hidden layer	1, 2
ANN layer number	64, 128

**Table 2:** Grid Search results for CNN structure.

Item	Rank				
	1	2	3	4	5
CNN channel	2-4-4-4	2-4-4-8	2-4-4-8	2-4-8-8	2-4-4-2
CNN filter step	2	2	2	2	1
CNN kernel	5	5	5	5	5
Max pool use	Not use	Not use	Not use	Not use	Use
ANN hidden layer	2	2	1	1	1
ANN layer number	128	64	128	64	128
ROM/RAM (Kbyte)	80.4/16.1	32/15.6	25.5/15.6	20.5/16.3	11.1/26.8
MAC	45.6 k	34.6 k	32.9 k	38.5 k	92.6 k
AVG ACC	99.72%	99.62%	99.54%	99.5%	99.49%

**Table 3:** Grid Search results for AE structure.

AE Zoom	AE Hidden Layer	ANN Hidden Layer	ANN Size
1.1	3	3	256

**Table 4:** Confusion matrix of online test results.

	True Class							Score	
	Load Shape	Large-Cylinder	Large-Square Column	Large-Hexagonal Column	Small-Cylinder	Small-Square Column	Small-Hexagonal Column	TPR	PRE
**Predicted Class**	large-cylinder	100	0	0	0	0	0	100%	99%
	large-square column	0	100	0	0	0	0	100%	100%
	large- hexagonal column	0	0	100	0	0	0	100%	100%
	small-cylinder	1	0	0	99	0	0	99%	100%
	small-square column	0	0	0	0	100	0	100%	100%
	small-hexagonal column	0	0	0	0	0	100	100%	100%
	ACC							99.83%	99.83%

**Table 5:** Confusion matrix of online test results for location variation.

	True Class							Score	
	Load Shape	Large-Cylinder	Large-Square Column	Large-Hexagonal Column	Small-Cylinder	Small-Square Column	Small-Hexagonal Column	TPR	PRE
**Predicted Class**	large-cylinder	177	0	3	0	0	0	98.3%	98.3%
	large-square column	0	180	0	0	0	0	100%	100%
	large-hexagonal column	2	0	175	0	0	3	97.2%	98.3%
	small-cylinder	1	0	0	170	0	10	94.4%	99.4%
	small-square column	0	0	0	1	176	3	97.7%	97.7%
	small-hexagonal column	0	0	0	0	4	176	97.7%	96.7%
	ACC							97.55%	98.4%

**Table 6:** Confusion matrix of online test results for classifying other objects.

	True Class								Score	
	Load Shape	Large-Cylinder	Large-Square Column	Large-Hexagonal Column	Small-Cylinder	Small-Square Column	Small-Hexagonal Column	Other Shape	TPR	PRE
**Predicted Class**	large-cylinder	50	0	0	0	0	0	0	100%	100%
	large-square column	0	50	0	0	0	0	0	100%	100%
	large-hexagonal column	0	0	49	0	0	0	0	98%	100%
	small-cylinder	0	0	0	49	0	0	0	98%	100%
	small-square column	0	0	0	0	48	0	0	96%	100%
	small-hexagonal column	0	0	0	0	0	49	0	98%	100%
	other shape	0	0	1	1	2	1	90	100%	94.7%
	ACC								98.5%	99.2%

**Table 7:** Confusion matrix of online test results for thicker objects.

	True Class								Score	
	Load Shape	Large-Cylinder	Large-Square Column	Large-Hexagonal Column	Small-Cylinder	Small-Square Column	Small-Hexagonal Column	Thicker Large-Cylinder	TPR	PRE
**Predicted Class**	large-cylinder	50	0	0	0	0	1	0	100%	98%
	large-square column	0	50	0	0	0	0	0	100%	100%
	large-hexagonal column	0	0	50	0	0	0	0	100%	100%
	small-cylinder	0	0	0	50	1	0	0	100%	98%
	small-square column	0	0	0	0	49	0	0	98%	100%
	small-hexagonal column	0	0	0	0	0	49	0	98%	100%
	thicker large-cylinder	0	0	0	0	0	0	50	100%	100%
	ACC								99.42%	99.42%

**Table 8:** Computation time of experiments.

Experiment	Sampling Time	STFT	AI Classification	Total
Experiment 1	0.100 s	0.092 s	0.160 s	0.352 s
Experiment 2			1.230 s	1.422 s
Experiment 3			CNN: 0.590 s AE: 0.036 s	0.818 s
